# Hospital Pharmacopœias

**Published:** 1905-01-07

**Authors:** 


### The Hospital.

A Journal of

The Medical Sciences and Hospital Administration.

Vol. XXXVII.—No. 954. JANUARY 7, 1905.

### Hospital Pharmacopœias.

The rush and pressure under which prescribing and dispensing are conducted in hospitals, and more especially in the out patient department, create a demand for time-saving arrangements which can hardly be resisted. One of these is the hospital pharmacopœia with its stated formulæ and ready-made prescriptions. The device is one which has obvious conveniences, and those who are familiar with all the circumstances will be the first to admit that under the conditions which at present exist it is in some measure inevitable. But this admission by no means justifies the hospital pharmacopœia in the form in which it is frequently found or overlooks the risks and evils which it entails. In many instances the volume is one of considerable size, and is carried into extremely elaborate detail. It is filled with prescriptions of greater or less value and antiquity, and even though subject to occasional revision, doubt may be entertained whether such work really engages the best attention of the hospital staff, or whether the finished product represents the highest result of modern therapeutical knowledge and skill. This is all the more serious seeing that, except in some occasional individual of independent mind, the pharmacopœia imposes itself on the members of the hospital staff, and defines the limits of their therapeutic methods and resources. The prescribing is apt as a consequence to become routine, and to be guided by the arrangements of the dispensary rather than by the demands of the patient. The effects of this evil are much exaggerated in hospitals associated with medical schools, where the pupils must either burden their memories with the composition of the several stock preparations, or must acquire the unfortunate habit of using names which are devoid of exact significance.

These evils might be met, at least in part, by the reduction of the hospital pharmacopœia to much simpler proportions. A relatively small number of formulæ might readily be constructed which could be used as bases to which other remedies might be added in harmony with the requirements of the individual case. Thus the practical demands of the moment would be met without serious invasion of the habit of scientific prescribing, and the reasonable claims of the dispenser and the medical student would receive adequate consideration.

Another unfortunate outcome of the hospital pharmacopœia as it exists to-day is the cultivation of the spirit of parochialism. The medical student grows up in the custom and habit of the sacred volume approved and adopted by his *alma mater*, and with a loyalty by no means unbecoming continues the tradition in his own mind and practice. Yet no one will deny that even the best of such pharmacopœias represents a very narrow range of therapeutic achievement, and the stereotyped habits they engender are far from the ideal to be desired for every practitioner. Moreover, the whole current of things is inclining towards the conversion of the present teaching hospitals into purely clinical schools, with the development of one or more central colleges for the teaching of the earlier scientific subjects and of anatomy and physiology. In such circumstances it is highly unlikely that each student will adhere throughout the whole of his training to the practice of a single hospital. He will be drawn here or there by the influence of a great teacher, or by special arrangements to meet his individual wants. The elaborate details and conflicting interpretations of numerous hospital pharmacopœias will, in this event, become an intolerable burden. The result, we fear, will be that the present tendency to pay inadequate attention to details of treatment will be exaggerated, and this cannot be otherwise than a public misfortune. It would be no slight service rendered to the cause of medical education and to sound medical practice were a properly constituted committee appointed to reduce the existing hospital pharmacopœias to the simplest elements permissible by the practical claims of hospital work, and out of these to compile a small volume which could be adopted in all public hospitals and dispensaries. The ideal system would be the entire abolition of routine prescribing and stock preparations and the substitution of a carefully devised prescription to meet the wants of each individual patient. But this, however much to be desired, is in present circumstances impossible. A considerable measure of reform on the lines we have indicated is by no means impossible, and were a movement in this direction taken seriously in hand the result would be conducive to wise and scientific practice. Many if not all hospital physicians much deplore the tyranny of their local pharmacopœia. They will serve the interests of their own freedom, of the cultivation of right therapeutic methods, and of the proper training of the rising generation of practitioners by demanding simplicity and uniformity in their officinal formulæ. The impending developments in medical education in London make the time apt for such a change, and we trust that some of our leading teachers will recognise the opportunity. In its present form the hospital pharmacopœia is an increasing evil. It is time it were reduced to a position more suitable to its comparative unimportance.

